# Supplementary material for: Asymptotically fault-tolerant programmable photonics
Source: Nat Commun. 2022 Nov 29;13:6831. doi: 10.1038/s41467-022-34308-3 (PMC9708693; doi:10.1038/s41467-022-34308-3)
Supplement: Supplementary file 1 — Supplementary Information [file 41467_2022_34308_MOESM1_ESM.pdf]

[Supplementary Material] Asymptotically Fault-Tolerant Programmable Photonics

Ryan Hamerly[1,2,*], Saumil Bandyopadhyay[1], Dirk Englund[1]

[1] *Research Laboratory of Electronics, MIT, 50 Vassar Street, Cambridge, MA 02139, USA*
[2] *NTT Research Inc., Physics and Informatics Laboratories, 940 Stewart Drive, Sunnyvale, CA 94085, USA*
[*] rhamerly@mit.edu

1 Error Correction Methods

Correction of hardware errors is performed using the nulling method, which is based on the diagonalization of a unitary matrix using Givens rotations. This is closely related to the QR decomposition for the Reck triangle [1], and a related decomposition for the more compact Clements rectangle [2]. The original nulling proposal was restricted to triangular (Reck) meshes and used internal tap detectors to monitor the output power of each MZI [3, 4]. Subsequently, the method was extended to generic mesh types [5], and Ref. [6] showed that that external detectors are sufficient for both Reck and Clements meshes.

Following Appendix A of Ref. [6], we describe here the nulling procedure for configuring a Reck mesh. First, we write the coupling matrix for the multiport interferometer as a product of the 2×2 MZI blocks and an external phase screen:

$$U = D\,\underbrace{\left(T_{N-1,1}\ldots T_{13}T_{12}T_{11}\right)}_{W} \qquad (1)$$

Here, the T_{mn} represent tunable couplers (MZI, 3-MZI, MZI+X, etc.) while D is a diagonal matrix encoding the output phase shifts. The T_{mn} are ordered along rising diagonals as shown in Fig. 1 (nulling also works on falling diagonals [6]).

Fig. 2 traces out the nulling steps for a 4×4 Reck mesh. We start by initializing the mesh to approximately the cross state, Fig. 2(a). We keep track of two matrices (Fig. 2(b)): $W = T_{N-1,1}\ldots T_{11}$ is the partial product of all configured MZIs, and $X = UW^\dagger$, where U is the target unitary. At the beginning, none of the MZIs are configured, so $W = I$ and $X = U$. At each step, with an example shown in Fig. 2(c), we configure target MZI T_{mn}, which updates W and X by Givens rotations $W \to T_{mn}W$, $X \to XT_{mn}^\dagger$. The target T_{mn} is chosen to zero a particular off-diagonal element X_{ij}. Subsequent MZIs are configured in a sequence that successively zeroes off-diagonal elements of X (Fig. 2(d)). If all MZIs are configured properly, at the end of the procedure, X is diagonalized so $U = DW$, and the output phases (elements of D) can be read off by inspection.

Nulling specifies constraints on the target Givens rotation T_{mn}, which zeroes an element of X by right-multiplication (Fig. 3). Assuming unitarity of all matrices, the zeroing

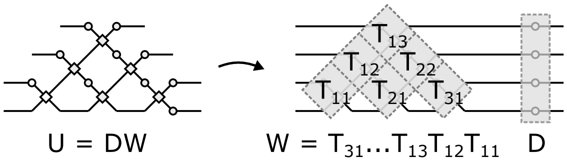

Figure 1: Reck decomposition of a 4×4 programmable unitary.

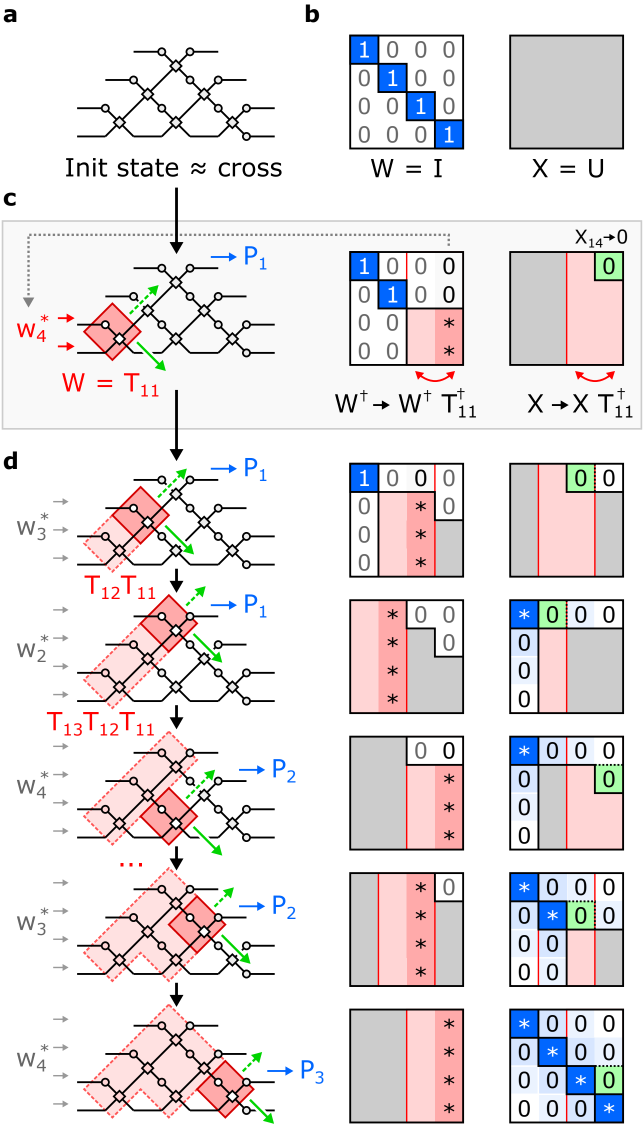

Figure 2: Configuration of a 4×4 Reck mesh by measurement-assisted nulling, following the procedure of Ref. [6].

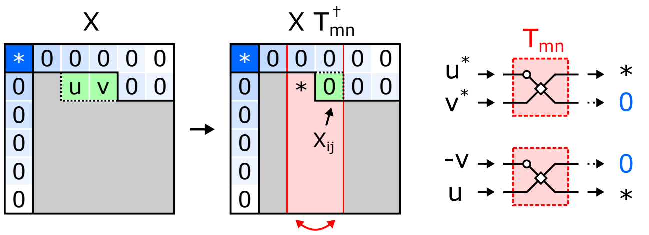

Figure 3: Right-multiplication by $T_m^\dagger$ mixes the elements (u, v) of X and zeroes out the rightmost one. This is equivalent to the condition Eq. (2).

of X_{ij} implies that:

$$T_{mn} \begin{bmatrix} -v \\ u \end{bmatrix} = \begin{bmatrix} 0 \\ * \end{bmatrix} \tag{2}$$

i.e. the power at the top output is zero when the fields $(-v, u)$ are input to the crossing. This is equivalent to the splitting-ratio condition $s = \hat{s}$, where $s \equiv (T_{mn})_{11}/(T_{mn})_{12}$ is the splitting ratio of the crossing (Eq. (2), main text), and $\hat{s} \equiv u/v$ is the target value. The difficulty in this procedure lies in the difficulty of accurately realizing $\hat{T}_{mn}$ in practice, since the actual transfer matrix is a function of both the control parameters (θ, ϕ) and the unknown fabrication imperfections. Therefore, for the *realized* Givens rotation, in general $s \neq \hat{s}$, which will lead to errors in the realized matrix U.

There are three distinct variants of the nulling method that accommodate hardware errors to different degrees: (1) an in-silico approach that does not correct errors [1, 2], (2) measurement-assisted nulling, which corrects errors provided that s does not fall within a forbidden region [3, 6], and (3) an improved measurement-assisted method that partially compensates for the "uncorrectable" errors arising from unrealizable splitting ratios.

1.1 In-Silico

Assuming ideal hardware, there is a simple relation between (θ, ϕ) and T. For example, for the standard MZI,

$$T = ie^{i\theta/2} \begin{bmatrix} e^{i\phi}\sin(\theta/2) & \cos(\theta/2) \\ e^{i\phi}\cos(\theta/2) & -\sin(\theta/2) \end{bmatrix} \tag{3}$$

in the absence of hardware errors. Using this formula, we can easily obtain (θ, ϕ) from the target splitting ratio:

$$\theta = 2\tan^{-1}|\hat{s}|, \quad \phi = \arg(\hat{s}) \tag{4}$$

Following this procedure, the phase shifts of the mesh are found entirely in a computer. As a result, hardware errors are not accounted for when programming the mesh, and the realized matrix will be off by an amount called the *uncorrected error*:

$$\mathcal{E}_0 \equiv \frac{\langle \|U - \hat{U}\| \rangle_{\text{rms}}}{\sqrt{N}} \approx \frac{1}{\sqrt{N}} \sqrt{\sum_{mn} \langle \|\Delta T_{mn}\|^2 \rangle} \tag{5}$$

where $\|\cdot\|$ is the Frobenius (L_2) norm, U and $\hat{U}$ are the realized and target matrices and $\Delta T_{mn} = T_{mn} - \hat{T}_{mn}$ is the difference (due to hardware errors) between the realized T_{mn} and the ideal $\hat{T}_{mn}$ given by Eq. (3).

In-silico methods were presented in Refs. [1, 2] for the Reck and Clements meshes. The effect of hardware errors was studied in Refs. [6, 7]. Fig. 4(a) shows the flowchart for programming a mesh via in-silico nulling.

1.2 Measurement-Assisted

In measurement assisted nulling, the first two steps are the same: find the target splitting ratio and update X and W using the corresponding Givens rotation. The principal difference is that (θ, ϕ) are found using an in-device measurement. For the Reck mesh, the procedure is traced out in Fig. 2, where each step attempts to zero a matrix element X_{ij} by injecting w_j^* as input and adjusting the phase shifters to zero the output power at port i (see also Fig. 4(b)). This method was first proposed [3] and demonstrated [4] on the Reck mesh, but can be generalized to other mesh types provided that tap detectors are present after every MZI [5]. It was later shown that self-configuration is possible without the tap detectors [6]. Errors occur whenever a crossing cannot be programmed to reach the target splitting ratio, i.e. when $\hat{s}$ lies within the forbidden region due to hardware imperfections.

1.3 Improved Measurement-Assisted

In this paper, we have developed a refinement to the measurement-assisted nulling algorithm that allows for some of the "uncorrectable" errors to be partially corrected in subsequent nulling steps. The impetus for this refinement is the observation that, whenever uncorrectable errors occur, the $s \neq \hat{s}$, and the conventional algorithm as implemented in Fig. 4(b) incorrectly updates X and W. Error correction can be improved if we can accurately estimate the realized splitting ratio s; this allows the algorithm to use this information in order to partially compensate for such errors during the programming of subsequent MZIs.

The refined error correction algorithm is shown in Fig. 4(c). Here, we defer updates to X and W until the end, and after (θ, ϕ) have been set, we measure s through the following procedure:

- If the output power is successfully nulled ($P_i = 0$), then the coupler is configured correctly and $s = \hat{s}$.
- If $P_i \neq 0$, nulling is imperfect and $s \neq \hat{s}$. To find s, we now perform an optimization: injecting $\bar{w}_j(s)$ (the jth column of $\bar{W}(s) = T(s)W$, which is a function of s), we vary s in the vicinity of $s = \hat{s}$ (with the fixed (θ, ϕ) obtained in the previous step) until the output power is exactly zero. This procedure obtains

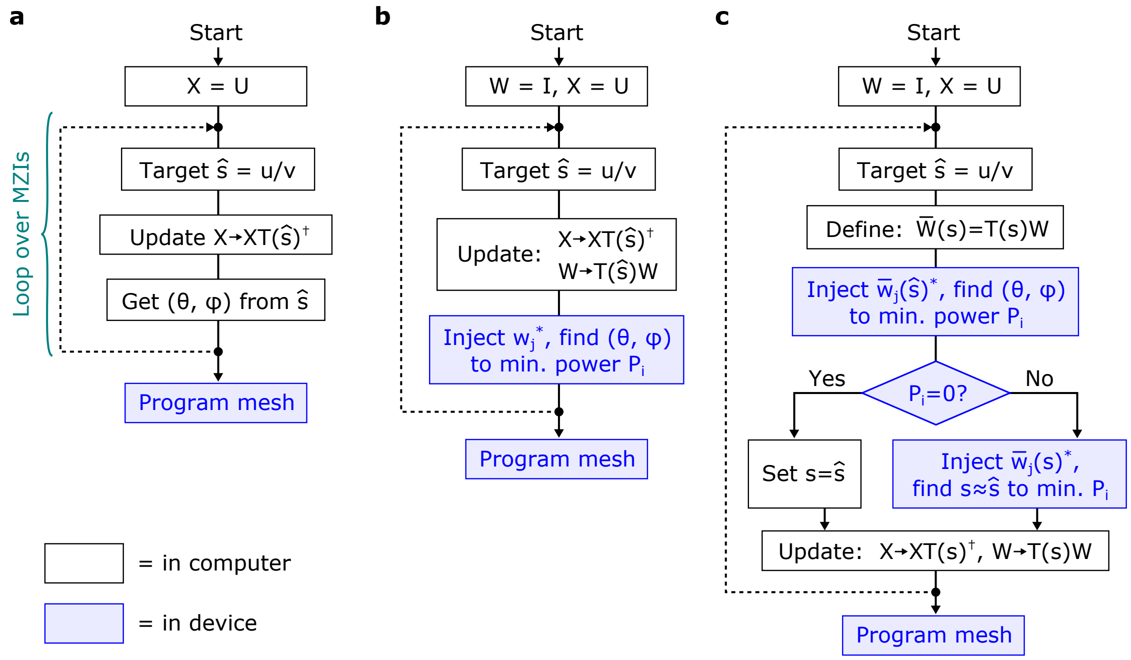

Figure 4: Nulling procedure for (a) in-silico (uncorrected) programming [1, 2], (b) measurement-assisted self-configuration [3, 6], and (c) an improvement to the measurement-assisted algorithm.

the actual splitting ratio implemented in the tunable coupler.

Once s is found, we update X and W using $T(s)$. Since the W and X updates are exact even in the presence of uncorrectable errors, the final matrix error is directly related to the residuals left by imperfect nulling of X. These residuals were calculated in the main text using the formula:

$$(\mathcal{E}_c^2)_{\rm sc} = \frac{2}{N}\sum_{mn}\langle r_{mn}^2\rangle = \frac{1}{2N}\sum_{mn}\underbrace{\langle |u_{mn}|^2+|v_{mn}|^2\rangle}_{q_{mn}} d(s_{mn},\hat{s}_{mn})^2 = \frac{\pi}{12N}\sum_{mn} q_{mn}\big[P_{mn}(s_+)\langle R_+^4\rangle + P_{mn}(s_-)\langle R_-^4\rangle\big] \quad (6)$$

1.4 Local Correction Method

For comparison, we also describe the local method for hardware error correction, first presented in Ref. [8]. This method is based on the principle that 2×2 unitary matrices are equivalent up to output phases if they share a common splitting ratio $s \equiv T_{11}/T_{12}$:

$$s = \hat{s} \quad\Leftrightarrow\quad T = \begin{bmatrix} e^{i\psi_1} & \\ & e^{i\psi_2}\end{bmatrix}\hat{T} \quad (7)$$

This equivalence principle allows perfect MZIs to be substituted for imperfect MZIs columnwise, performing correction locally at each coupler (although the procedure is not strictly local: each step depends on the phases ψ_i accrued from Eq. (7) in the previous step). Errors occur only when MZI splitting ratios are unrealizable. These "uncorrectable errors" are independent of each other and add up in quadrature. Refs. [6, 7] calculate the resulting matrix error, which follows from the relation

$$\|\Delta T\| \equiv \min_\psi \left\| T - \begin{bmatrix} e^{i\psi_1} & \\ & e^{i\psi_2}\end{bmatrix}\hat{T}\right\| = \frac{d(s,\hat{s})}{\sqrt{2}} \quad (8)$$

where $d(s,\hat{s}) = 2|s-\hat{s}|/\sqrt{(|s|^2+1)(|\hat{s}|^2+1)}$ is the Euclidean metric on the Riemann sphere (under the stereographic projection $s=(x+iy)/(1+z)$, which inverts to $x+iy = 2s/(1+|s|^2)$ and $z=(1-|s|^2)/(1+|s|^2)$).

In the notation of this paper, $\mathcal{E}_c$ takes the form:

$$(\mathcal{E}_c^2)_{\rm loc} \equiv \sum_{mn}\|\Delta T_{mn}\|^2 = \frac{1}{2N}\sum_{mn} d(s_{mn},\hat{s}_{mn})^2 = \frac{\pi}{12N}\sum_{mn}\big[P_{mn}(s_+)\langle R_+^4\rangle + P_{mn}(s_-)\langle R_-^4\rangle\big] \quad (9)$$

Eqs. (6) and (9) are almost identical, differing only by the factor of $q_{mn} = \langle |u_{mn}|^2 + |v_{mn}|^2\rangle$ in the former. Since $q_{mn} \leq 1$ due to the unitarity of X, Eq. (6) will always give a lower matrix error.

Table 1 lists the formulas for coverage (Eq. (13), main text) and matrix error (Eqs. (5-6)) for the three mesh architectures and error models. We see that, for uncorrelated errors, only the 3-MZI is asymptotically perfect, while both the 3-MZI and MZI+X are asymptotically perfect for correlated errors. In addition, the uncorrected

Model	Arch	Coverage $\mathcal{C}$	Matrix Error $\mathcal{E}_0^2$	$(\mathcal{E}_c^2)_{\text{loc}}$	$(\mathcal{E}_c^2)_{\text{sc}}$
(any)	MZI	$e^{-N^3\langle R_+^2\rangle/24-N\langle R_-^2\rangle/4}$	Eq. (5)	$\frac{N^2}{288}\langle R_+^4\rangle+\frac{1}{48}\langle R_-^4\rangle$	$\frac{N^2}{432}\langle R_+^4\rangle+\frac{\log N-0.422}{24N}\langle R_-^4\rangle$
	3-MZI	$e^{-N(\langle R_+^2\rangle+\langle R_-^2\rangle)}$		$\frac{1}{12}(\langle R_+^4\rangle+\langle R_-^4\rangle)$	$\frac{\log N-1.366}{3N}(\langle R_+^4\rangle+\langle R_-^4\rangle)$
	MZI+X	$e^{-N\langle R_+^2\rangle/4-N^3\langle R_-^2\rangle/24}$		$\frac{1}{48}\langle R_+^4\rangle+\frac{N^2}{288}\langle R_-^4\rangle$	$\frac{\log N-0.422}{24N}\langle R_+^4\rangle+\frac{N^2}{432}\langle R_-^4\rangle$
σ	MZI	$e^{-N^3\sigma^2/3}$	$2N\sigma^2$	$\frac{2}{3}N^2\sigma^4$	$\frac{4}{9}N^2\sigma^4$
	3-MZI	$e^{-16N\sigma^2}$	$3N\sigma^2$	$32\sigma^4$	$128\frac{\log(N)-1.366}{N}\sigma^4$
	MZI+X	$e^{-N^3\sigma^2/3}$	$N(2\sigma^2+\sigma_\gamma^2)$	$4\sigma^4$	$\frac{4}{9}N^2\sigma^4$
μ	MZI	$e^{-(2/3)N^3\mu^2}$	$4N\mu^2$	$\frac{8}{9}N^2\mu^4$	$\frac{16}{27}N^2\mu^4$
	3-MZI	$e^{-16N\mu^2}$	$3N\mu^2$	$\frac{32}{3}\mu^4$	$\frac{256}{3}\frac{\log N-1.366}{N}\mu^4$
	MZI+X	$e^{-4N\mu^2}$	$8(\log(N)-1.422)\mu^2$	$\frac{16}{3}\mu^4$	$\frac{32}{3}\frac{\log N-0.422}{N}\mu^4$
$\mu \gg \sigma$	(any)	$\mathcal{C}=\mathcal{C}_\mu\times\mathcal{C}_\sigma$	$\mathcal{E}^2=\mathcal{E}_\mu^2+\mathcal{E}_\sigma^2$		

Table 1: Coverage and matrix error for the MZI, 3-MZI, and MZI+X designs. Matrix error is given for the three nulling methods: in silico (uncorrected, Sec. 1.1), local correction (Sec. 1.4) and self-configuration (Sec. 1.3). While the error formulas are general, specific results are given for the uncorrelated model (σ) and the perfectly correlated model (μ).

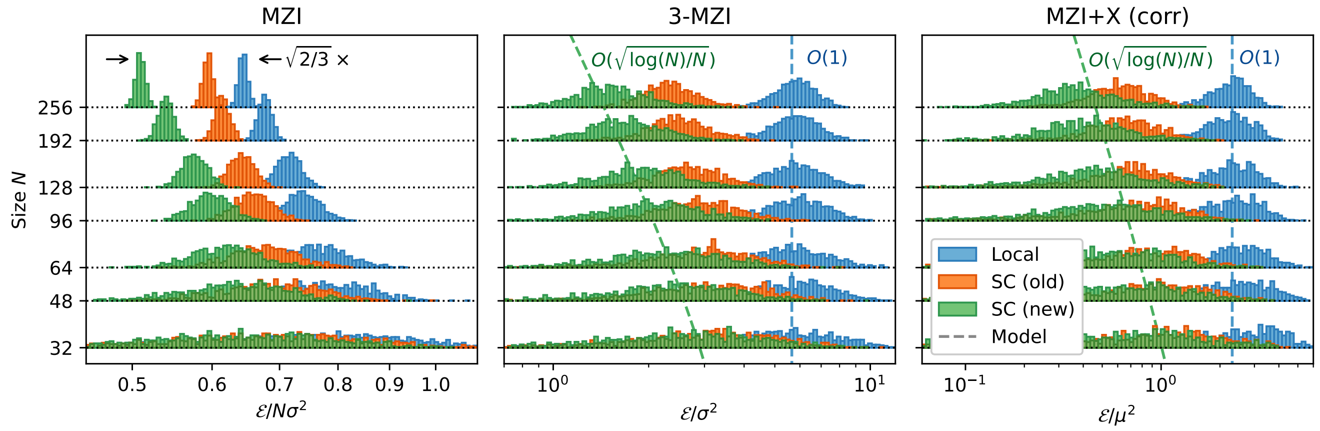

Figure 5: Comparison of the accuracy of self-configuration (SC, Sec. 1.2-1.3) and the local error correction method, Sec. 1.4. For the MZI and 3-MZI, Gaussian error models are used with $\sigma = 0.05$ and $\sigma = 0.10$, respectively. For the MZI+X, a correlated error model with $\mu = 0.1$ is used. Dashed lines correspond to the analytic models in Table 1.

error can only be reduced in the correlated case, and only for the MZI+X. Finally, the examples of the 3-MZI and MZI+X highlight the superior performance of the improved self-configuration method. Under the original method, $\mathcal{E}_c$ is independent of N, making the mesh types infinitely scalable (with respect to these errors) but not asymptotically perfect. But under the improved method, $\mathcal{E}_c \propto \sqrt{\log(N)/N}$, which vanishes in the limit $N \to \infty$.

Fig. 5 plots the numerically computed accuracy on the three mesh types. For the 3-MZI and MZI+X, the difference in scaling with N is very clear. For the regular MZI, all methods give the same scaling, but self-configuration leads to an error lower by a factor of $\sqrt{2/3}$ ($\sqrt{2/3}N\sigma^2$ vs. $(2/3)N\sigma^2$). The overall error amplitude in the figure is distorted by saturation when $\mathcal{E} \sim 1$, but the factor of $\sqrt{2/3}$ is still clearly apparent.

1.5 Comparison to Global Optimization

Before the self-configuration and local algorithms were developed, the only way to train imperfect meshes involved global optimization [9–11]. Since meshes are linear and reciprocal devices, backpropagation of gradients is equivalent to traversing the mesh in the opposite direction [12]. This is implemented in most simulation packages, including Neurophox [13] (based on PyTorch backend) and Meshes [14] (based on NumPy with Numba/CUDA extensions), and leads to optimization times orders of magnitude shorter than gradient-free methods.

Previous studies have shown that gradient-based optimization can give a slight improvement in the matrix fidelity compared to the local or self-configuration approaches [6, 7], but take significantly longer to run, in practice requiring thousands of iterations to converge to a

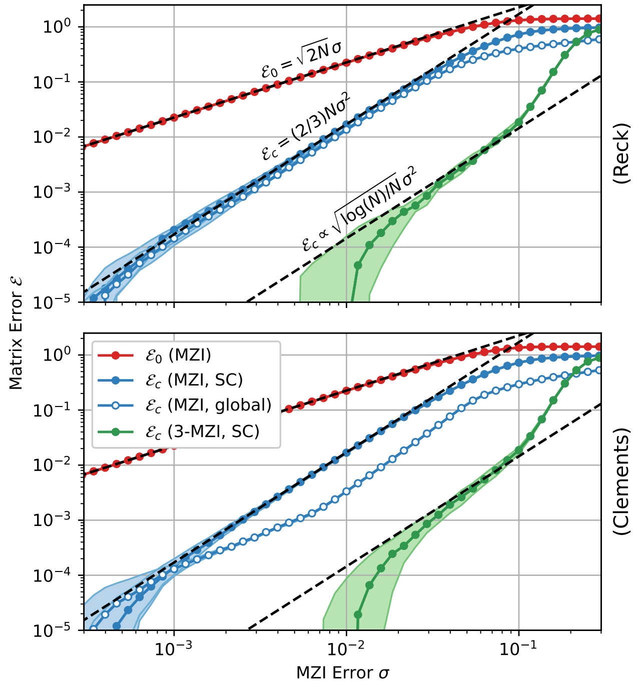

Figure 6: Comparison of self-configuration and global optimization for $N = 256$ meshes of the Reck (top) and Clements (bottom) topology, with uncorrelated errors.

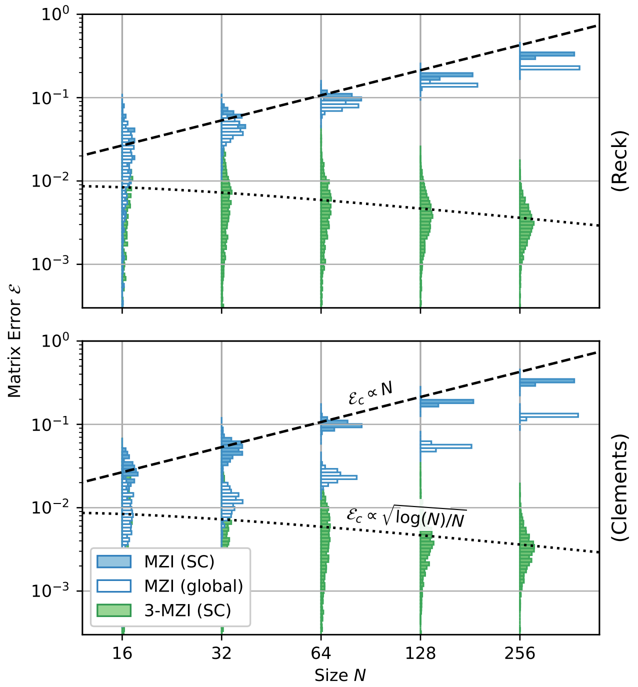

Figure 7: Dependence of corrected matrix error $\mathcal{E}_c$ on mesh size. Model: uncorrelated splitter errors with $\sigma = 0.05$.

solution that is non-negligibly better than the algorithms of Sec. 1.3. However, given sufficient computation time, refinement by global optimization may be an appropriate error correction technique. Using the GPU backend of Meshes, we performed gradient-based optimization on faulty meshes, using the L-BFGS-B algorithm and the self-configured solution as an initial condition. Figs. 6-7 compare the accuracy of the self-configured solution to this global refinement. Interestingly, the improvement is fairly significant (3–4×) for Clements, but negligible for Reck. We speculate that this discrepancy may be attributed to the triangular structure of Reck, where the MZIs near the apex of the triangle are most likely to lead to uncorrectable errors. Since the upper-left corner of the matrix depends only on these MZIs, errors in this region cannot be corrected by adjustments to MZIs up- or downstream. This is in contrast to the Clements mesh, where all paths pass through an equal number of MZIs, and errors in the center of the mesh (where the probability density clusters close to the cross state) can potentially be corrected by adjustments near the edges.

In both meshes, up to a constant factor, the self-configured and globally-optimized solutions have the same error scaling $\mathcal{E}_c \propto N\sigma^2$ in the MZI mesh. For large mesh sizes, the 3-MZI mesh still offers a significant improvement over the globally optimized solutions, and its $\mathcal{E}_c \propto \sqrt{\log(N)/N}$ scaling means that this gap grows larger with increasing mesh size.

2 Imperfectly Correlated Errors

The splitter errors (α, β) of an MZI are best characterized by measuring the device's extinction ratio. To do so, one tunes the internal phase shifter θ and measures the contrast of the interference fringes on the bar- and cross-port outputs. As an MZI has the following transfer matrix

$$T(\theta,\phi) = \begin{bmatrix} \cos(\frac{\pi}{4}+\beta) & i\sin(\frac{\pi}{4}+\beta) \\ i\sin(\frac{\pi}{4}+\beta) & \cos(\frac{\pi}{4}+\beta) \end{bmatrix} \begin{bmatrix} e^{i\theta} & 0 \\ 0 & 1 \end{bmatrix} \times \begin{bmatrix} \cos(\frac{\pi}{4}+\alpha) & i\sin(\frac{\pi}{4}+\alpha) \\ i\sin(\frac{\pi}{4}+\alpha) & \cos(\frac{\pi}{4}+\alpha) \end{bmatrix} \begin{bmatrix} e^{i\phi} & 0 \\ 0 & 1 \end{bmatrix} \quad (10)$$

the bar- and cross-port outputs are have extrema $\theta \in \{0, \pi\}$. The and the extinction ratios are given by:

$$\mathrm{ER_{bar}[dB]} = 20\log_{10}\left|\frac{T_{11}(\theta=\pi)}{T_{11}(\theta=0)}\right| = 20\log_{10}\left|\frac{\cos(\alpha-\beta)}{\sin(\alpha+\beta)}\right| \approx -20\log_{10}|\alpha+\beta| \quad (11)$$

$$\mathrm{ER_{cross}[dB]} = 20\log_{10}\left|\frac{T_{21}(\theta=\pi)}{T_{21}(\theta=0)}\right| = 20\log_{10}\left|\frac{\cos(\alpha+\beta)}{\sin(\alpha-\beta)}\right| \approx -20\log_{10}|\alpha-\beta| \quad (12)$$

These relations can be inverted to give us:

$$|\alpha+\beta| = 10^{-\mathrm{ER_{bar}}/20}, \quad |\alpha-\beta| = 10^{-\mathrm{ER_{cross}}/20} \quad (13)$$

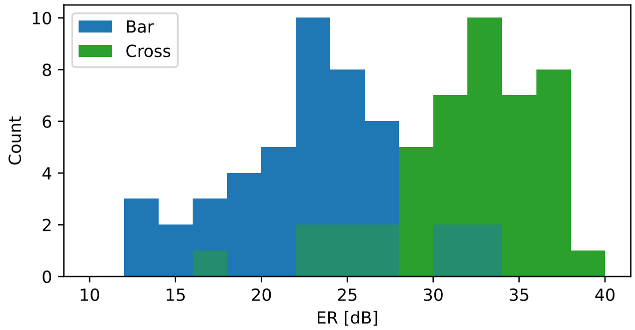

Figure 8: Distribution of extinction ratios for MZIs characterized in 3-layer neural network chip of Ref. [8].

Ref	Type	Platform	ER_{cross}	ER_{bar}
[18]	MZI	SiO_2 PLC	29	–
[19]	MZI	SiO_2 PLC	25.9	–
[20]	MZI	SiO_2 PLC	32.5	–
[21]	MZI	SiO_2 PLC	31	22
[22]	MZI	SOI	35	25
[23]	MZI	SOI	34	–
[24]	MZI	SOI	34	35
[25]	MZI	SOI	41.2	–
[26]	MZI	SOI	–	30.9
[27]	MZI	SiN:AlN	30	–
[28]	Suzuki	SOI	50.4	
[26]	Miller	SOI	60.5	

Table 2: Reported bar- and cross-port MZI extinction ratios. Extinction ratios are reported in dB.

In most photonic platforms, splitter errors are strongly correlated so that $ER_{cross} \gg ER_{bar}$. For example, in Fig. 8, we plot a histogram of measured MZI extinction ratios characterized for a 3-layer silicon-photonic neural network chip reported in Ref. [8]. The median bar- and cross-port extinction ratios are 23 dB and 32 dB, respectively. This correlation between splitter errors originates from the lengthscales of fabrication process variations that affect the critical dimensions (width, height spacing) of the directional couplers. These variations typically have correlation lengths on the order of millimeters [15–17], significantly longer than the spacing between couplers in an MZI. This trend is also observed elsewhere in the literature, as shown in Table 2. This suggests an imperfectly-correlated error model of the form

	MZI	3-MZI	MZI+X
s_+	0	i	∞
s_-	∞	$-i$	0

Table 3: Locations of the forbidden regions for each mesh crossing geometry.

$$\alpha \sim N(\mu, \sigma), \quad \beta \sim N(\mu, \sigma) \tag{14}$$

with $\mu \gg \sigma$, is most accurate. We can use Eq. (13) to relate μ and σ to the median MZI extinction ratios, as follows:

$$\mu = \frac{10^{-\mathrm{ER_{bar}}/20}}{2}, \quad \sigma = \frac{10^{-\mathrm{ER_{cross}}/20}}{2.10} \tag{15}$$

Recall that the Riemann sphere has two forbidden regions centered at $s_\pm$ (see Table 3) with radii $R_\pm = 2|\alpha \pm \beta|$. Under this model, $R_\pm$ has the following moments:

$$\begin{aligned} \langle R_+^2\rangle &= 16\mu^2\big(1+\tfrac{1}{2}(\sigma/\mu)^2\big) & \langle R_-^2\rangle &= 8\sigma^2 \\ \langle R_+^4\rangle &= 256\mu^4\big(1+3(\sigma/\mu)^2+\tfrac{3}{4}(\sigma/\mu)^4\big) & \langle R_-^4\rangle &= 192\sigma^4 \end{aligned} \tag{16}$$

The locations of the forbidden regions are given in Table 3. Following the derivation in the Methods (specifically Eqs. (14, 19, 21, 26)), we find:

$$(\mathcal{E}_c)^2 = \begin{cases} \dfrac{N^2}{432}\langle R_+^4\rangle + \dfrac{\log(N)-0.422}{24N}\langle R_-^4\rangle & \text{(MZI)} \\ \dfrac{\log(N)-1.366}{3N}\big(\langle R_+^4\rangle + \langle R_-^4\rangle\big) & \text{(3-MZI)} \\ \dfrac{\log(N)-0.422}{24N}\langle R_+^4\rangle + \dfrac{N^2}{432}\langle R_-^4\rangle & \text{(MZI+X)} \end{cases} \tag{17}$$

where we have substituted $(\frac{5}{4}+\log(2)-\gamma_e) \to 1.366$ and $(1-\gamma_e) \to 0.422$ for clarity.

In the main text, we considered the special cases (1) *Uncorrelated errors*, $\mu = 0$, where Eqs. (17) reduces to Eqs. (3, 14, 22) (main text), and (2) *Perfectly correlated errors*, $\sigma = 0$, where Eqs. (17) reduces to Eqs. (4, 27) (main text). Here we consider the imperfectly correlated case, where $\sigma \ll \mu$. Substituting Eqs. (16) into Eqs. (17) and only keeping terms leading order in (σ/μ), we find:

$$\mathcal{E}_c = \begin{cases} \dfrac{4}{3^{3/2}}N\mu^2 & \text{(MZI)} \\ \dfrac{16\mu^2}{\sqrt{3}}\Big[\dfrac{\log(N)-1.366}{N}\Big]^{1/2} & \text{(3-MZI)} \\ \Big[\dfrac{32\mu^4}{3}\dfrac{\log(N)-0.422}{N} + \big((2/3)N\sigma^2\big)^2\Big]^{1/2} & \text{(MZI+X)} \end{cases} \tag{18}$$

For the MZI and 3-MZI, the error is determined entirely by the mean value μ. On the other hand, for the MZI+X design, the scaling with N in Eq. (18) means that the accuracy of large meshes is limited by the differential term σ even though $\sigma \ll \mu$. This is shown in Fig. 9, which shows the effect of nonzero σ (characterized in terms of the cross-port ER through Eq. (15)) on the MZI+X mesh. We see that these small differential errors ultimately limit the scaling of this mesh, which is only asymptotically

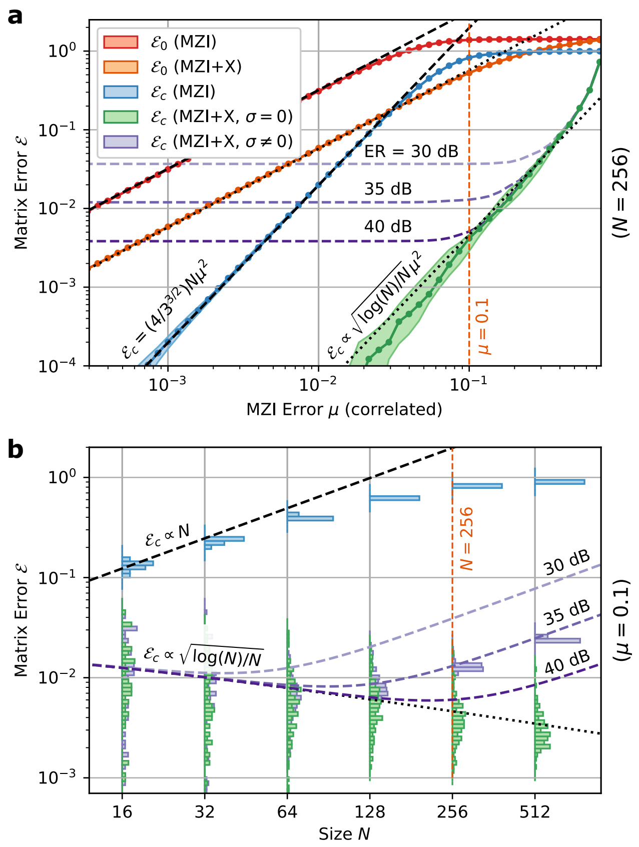

Figure 9: Effect of finite cross-port extinction ratio on corrected error for MZI+X; compare Fig. 6 (main text). (a) Dependence of matrix error on μ for a Reck mesh of fixed size $N = 256$. (b) Dependence on N for fixed $\mu = 0.1$.

perfect in the ideal case of perfectly correlated errors. However, for a reasonable value of $\text{ER}_{\text{cross}} = 35$ dB (see Table 2), $\mathcal{E}_c$ is at most a few percent for mesh sizes up to $N = 512$. This suggests that error correction allows the MZI+X to be asymptotically perfect on all practical mesh sizes, as scaling to meshes of size $N > 512$ is likely prohibitively challenging due to chip area and loss constraints.

In order to exactly cancel the differential term $\alpha - \beta$ as required for very large meshes $N > 1024$, one can place a heater above the directional coupler [29]. While this scheme does come with the cost of an additional active component (putting it in the same complexity category as the "perfect optics" approaches [28, 30]), such an MZI+X with coupler trimming is unique in that it enjoys natively broad bandwidth, enhancing the WDM capacity of the system, which may prove critical to achieving competitive performance in photonic computing applications [31].

3 Length and Area Estimates

Table 2 of the main text provides a rough comparison of the resource costs of various mesh architectures. In all cases, the "perfect optics" designs [28, 30] require 1.5–2× more active components, an important near-term concern as the size of existing chips is often limited by electronic packaging [35] or power dissipation from heaters [36]. Waveguide length (which limits loss and SNR [37] and on-chip latency [8, 38]) and chip area are also critical parameters, but depend on the implementation.

The approximate MZI dimensions of a range of photonic mesh platforms are reported in Table 4. Most SOI devices has similar sizes, although there is a wider range of phase-shifter lengths owing to design tradeoffs (longer thermo-optic phase shifters can be more energy-efficient in certain cases [39] and the higher heater resistance reduces the required current, but such devices suffer from increased loss and/or higher drive voltages). Non-SOI platforms such as silicon nitride and lithium niobate can support shorter optical wavelengths and offer mechanisms for faster pure-phase modulation, but suffer from reduced integration density due to the weaker phase-shift mechanisms (e.g. Pockels [34] or piezo-optomechanical [27]), which require much longer phase shifters. In such platforms, the length and area reduction for the 3-MZI is particularly pronounced, as these figures depend primarily on the number of phase shifters and not the number of passive components.

References

[1] Reck, M., Zeilinger, A., Bernstein, H. J. & Bertani, P. Experimental realization of any discrete unitary operator. *Physical Review Letters* **73**, 58 (1994).

[2] Clements, W. R., Humphreys, P. C., Metcalf, B. J., Kolthammer, W. S. & Walmsley, I. A. Optimal design for universal multiport interferometers. *Optica* **3**, 1460–1465 (2016).

[3] Miller, D. A. Self-configuring universal linear optical component. *Photonics Research* **1**, 1–15 (2013).

[4] Annoni, A. *et al.* Unscrambling light—automatically undoing strong mixing between modes. *Light: Science & Applications* **6**, e17110–e17110 (2017).

[5] Miller, D. A. Setting up meshes of interferometers–reversed local light interference method. *Optics Express* **25**, 29233–29248 (2017).

[6] Hamerly, R., Bandyopadhyay, S. & Englund, D. Accurate self-configuration of rectangular multiport interferometers. *Physical Review Applied* **18**, 024019 (2022).

Ref	Platform	**WG** [μm]		**Dimensions** [μm]			**Length Multiplier**			**Area Multiplier**		
		ℓ_{ph}	ℓ_{bs}	w_{ph}	w_{bs}	h	3-MZI†	Suzuki	Miller	3-MZI†	Suzuki	Miller
[23]	SOI	80	170	80	100	140	1.34×	1.5×	2.0×	1.27×	1.5×	2.0×
[25]	SOI	80	180	80	80	200	1.35×	1.5×	2.0×	1.25×	1.5×	2.0×
[28]	SOI	180	200	180	90	180	1.26×	1.5×	2.0×	1.17×	1.5×	2.0×
[32]	SOI	950	130	250	90	130	1.06×	1.5×	2.0×	1.13×	1.5×	2.0×
[26]	SOI	200	220	200	125	200	1.26×	1.5×	2.0×	1.19×	1.5×	2.0×
[8]	SOI	200	160	200	80	150	1.22×	1.5×	2.0×	1.14×	1.5×	2.0×
[33]	SiN	1300	400	1300	400	300	1.12×	1.5×	2.0×	1.12×	1.5×	2.0×
[27]	SiN:AlN	10^4	10^3	200	100	1200	1.05×	1.5×	2.0×	1.17×	1.5×	2.0×
[34]	$LiNbO_3$	10^4	10^3	10^4	10^3	100	1.05×	1.5×	2.0×	1.05×	1.5×	2.0×

Table 4: Waveguide (WG) length ℓ and on-chip areal dimensions ($w \times h$) of phase shifters and beamsplitters on several published photonic platforms. The corresponding unit-cell length and area $A = wh$ (normalized to the standard MZI) are computed from these dimensions. †MZI+X will have a size similar to 3-MZI.

[7] Hamerly, R., Bandyopadhyay, S. & Englund, D. Stability of self-configuring large multiport interferometers. *Physical Review Applied* **18**, 024018 (2022).

[8] Bandyopadhyay, S. *et al.* Single chip photonic deep neural network with accelerated training. *arXiv preprint arXiv:2208.01623* (2022).

[9] Mower, J., Harris, N. C., Steinbrecher, G. R., Lahini, Y. & Englund, D. High-fidelity quantum state evolution in imperfect photonic integrated circuits. *Physical Review A* **92**, 032322 (2015).

[10] Burgwal, R. *et al.* Using an imperfect photonic network to implement random unitaries. *Optics Express* **25**, 28236–28245 (2017).

[11] Pai, S., Bartlett, B., Solgaard, O. & Miller, D. A. Matrix optimization on universal unitary photonic devices. *Physical Review Applied* **11**, 064044 (2019).

[12] Hughes, T. W., Minkov, M., Shi, Y. & Fan, S. Training of photonic neural networks through in situ backpropagation and gradient measurement. *Optica* **5**, 864–871 (2018).

[13] Pai, S. Neurophox: a simulation framework for unitary neural networks and photonic devices. Online at: `https://github.com/solgaardlab/neurophox` (2020).

[14] Hamerly, R. Meshes: tools for modeling photonic beamsplitter mesh networks. Online at: `https://github.com/QPG-MIT/meshes` (2021).

[15] Yang, Y. *et al.* Phase coherence length in silicon photonic platform. *Optics Express* **23**, 16890–16902 (2015).

[16] Chrostowski, L. *et al.* Impact of fabrication non-uniformity on chip-scale silicon photonic integrated circuits. In *Optical Fiber Communication Conference*, Th2A–37 (Optical Society of America, 2014).

[17] Bogaerts, W., Xing, Y. & Khan, U. Layout-aware variability analysis, yield prediction, and optimization in photonic integrated circuits. *IEEE Journal of Selected Topics in Quantum Electronics* **25**, 1–13 (2019).

[18] Kawachi, M. *et al.* Silica-based optical-matrix switch with intersecting Mach-Zehnder waveguides for larger fabrication tolerances. In *Optical Fiber Communication Conference*, TuH4 (Optical Society of America, 1993).

[19] Nagase, R. *et al.* Silica-based 8×8 optical matrix switch module with hybrid integrated driving circuits and its system application. *Journal of Lightwave Technology* **12**, 1631–1639 (1994).

[20] Goh, T., Himeno, A., Okuno, M., Takahashi, H. & Hattori, K. High-extinction ratio and low-loss silica-based 88 strictly nonblocking thermooptic matrix switch. *Journal of Lightwave Technology* **17**, 1192 (1999).

[21] Okuno, M. *et al.* Silica-based 8×8 optical matrix switch integrating new switching units with large fabrication tolerance. *Journal of Lightwave Technology* **17**, 771–781 (1999).

[22] Shoji, Y. *et al.* Low-crosstalk 2×2 thermo-optic switch with silicon wire waveguides. *Optics Express* **18**, 9071–9075 (2010).

[23] Harris, N. C. *et al.* Quantum transport simulations in a programmable nanophotonic processor. *Nature Photonics* **11**, 447–452 (2017).

[24] Dumais, P. *et al.* Silicon photonic switch subsystem with 900 monolithically integrated calibration photodiodes and 64-fiber package. *Journal of Lightwave Technology* **36**, 233–238 (2017).

[25] Suzuki, K. *et al.* Low-insertion-loss and power-efficient 32×32 silicon photonics switch with ex-

tremely high-δ silica PLC connector. *Journal of Lightwave Technology* **37**, 116–122 (2018).

[26] Wilkes, C. M. *et al.* 60 dB high-extinction auto-configured Mach-Zehnder interferometer. *Optics Letters* **41**, 5318–5321 (2016).

[27] Dong, M. *et al.* High-speed programmable photonic circuits in a cryogenically compatible, visible–near-infrared 200 mm CMOS architecture. *Nature Photonics* **16**, 59–65 (2022).

[28] Suzuki, K. *et al.* Ultra-high-extinction-ratio 2×2 silicon optical switch with variable splitter. *Optics Express* **23**, 9086–9092 (2015).

[29] Orlandi, P. *et al.* Tunable silicon photonics directional coupler driven by a transverse temperature gradient. *Optics Letters* **38**, 863–865 (2013).

[30] Miller, D. A. Perfect optics with imperfect components. *Optica* **2**, 747–750 (2015).

[31] Feldmann, J. *et al.* Parallel convolutional processing using an integrated photonic tensor core. *Nature* **589**, 52–58 (2021).

[32] Wang, M., Ribero, A., Xing, Y. & Bogaerts, W. Tolerant, broadband tunable 2×2 coupler circuit. *Optics Express* **28**, 5555–5566 (2020).

[33] Taballione, C. *et al.* 20-mode universal quantum photonic processor. *arXiv preprint arXiv:2203.01801* (2022).

[34] Wu, R. *et al.* Fabrication of a multifunctional photonic integrated chip on lithium niobate on insulator using femtosecond laser-assisted chemomechanical polish. *Optics Letters* **44**, 4698–4701 (2019).

[35] Siew, S. Y. *et al.* Review of silicon photonics technology and platform development. *Journal of Lightwave Technology* **39**, 4374–4389 (2021).

[36] Kumar, S. P. *et al.* Mitigating linear optics imperfections via port allocation and compilation. *arXiv preprint arXiv:2103.03183* (2021).

[37] Al-Qadasi, M., Chrostowski, L., Shastri, B. & Shekhar, S. Scaling up silicon photonic-based accelerators: Challenges and opportunities. *APL Photonics* **7**, 020902 (2022).

[38] Shen, Y. *et al.* Deep learning with coherent nanophotonic circuits. *Nature Photonics* **11**, 441 (2017).

[39] Qiu, H. *et al.* Energy-efficient thermo-optic silicon phase shifter with well-balanced overall performance. *Optics Letters* **45**, 4806–4809 (2020).
